# Lipoxin A4 Regulates Lipopolysaccharide-Induced BV2 Microglial Activation and Differentiation via the Notch Signaling Pathway

**DOI:** 10.3389/fncel.2019.00019

**Published:** 2019-02-04

**Authors:** Jun Wu, Dan-hua Ding, Qian-qian Li, Xin-yu Wang, Yu-ying Sun, Lan-Jun Li

**Affiliations:** Department of Neurology, The First Affiliated Hospital of Zhengzhou University, Zhengzhou, China

**Keywords:** inflammatory response, microglia, lipopolysaccharide, LXA4, Notch signaling pathway

## Abstract

Inflammatory responses contribute to the pathogenesis of various neurological diseases, and microglia plays an important role in the process. Activated microglia can differentiate into the pro-inflammatory, tissue-damaging M1 phenotype or the anti-inflammatory, tissue-repairing M2 phenotype. Regulating microglia differentiation, hence limiting a harmful response, might help improve the prognosis of inflammation-related nervous system diseases. The present study aimed 1. to observe the anti-inflammatory effect of lipoxin A4 (LXA4) on the inflammatory response associated to lipopolysaccharide (LPS)-induced microglia activation, 2. to clarify that LXA4 modulates the activation and differentiation of microglia induced by LPS stimulation, 3. to determine whether LXA4 regulates the activation and differentiation of microglia through the Notch signaling pathway, 4. to provide a foundation for the use of LXA4 for the treatment of inflammatory related neurological diseases. To construct a model of cellular inflammation, immortalized murine BV2 microglia cells were provided 200 ng/ml LPS. To measure the mRNA and protein levels of inflammatory factors (interleukin [IL]-1β, IL-10, and tumor necrosis factor [TNF]-α) and M1 and M2 microglia markers (inducible nitric oxide synthase [iNOS], cluster of differentiation [CD]32, arginase [Arg]1, and CD206), we performed quantitative reverse transcription polymerase chain reaction (qRT-PCR) and enzyme-linked immunosorbent assay (ELISA), immunofluorescence, or flow cytometry. To determine the mRNA and protein levels of Notch signaling components (Notch1, Hes1, and Hes5), we performed qRT-PCR and western blot. LXA4 inhibits the expression of Notch1 and Hes1 associated with M1 type microglial differentiation and decreases the M1 type microglia marker iNOS and related inflammatory factors IL-1β and TNF-α. Moreover, LXA4 upregulates the expression of the M2-associated Hes5, as well as the expression of the M2 microglia marker Arg1 and the associated inflammatory factor IL-10. These effects are blocked by the administration of the γ-secretase inhibitor DAPT, a specific blocker of the Notch signaling pathway. LXA4 inhibits the microglia activation induced by LPS and the differentiation into M1 type with pro-inflammatory effect, while promoting the differentiation to M2 type with anti-inflammatory effect. LXA4 downregulates the inflammatory mediators IL-1β, TNF-α, and iNOS, while upregulating the anti-inflammatory mediator IL-10, which acts through the Notch signaling pathway.

## Introduction

Numerous studies have shown that neuroinflammation plays an important role in the occurrence and development of central nervous system disorders such as ischemic stroke, Alzheimer’s disease, and Parkinson’s disease, and is associated to every stage of the disease process(Griffin, 2006; McColl et al., 2009; Naegele and Martin, 2014; Wang Q. et al., 2015; Cuello, 2017). The inflammatory response is an automatic defense response of the body to stimuli, which can promote the clearance of pathogenic factors and the healing of damaged tissue. It is usually beneficial, but it is a double-edged sword and is harmful in some cases. For example, the inflammatory response of the central nervous system sometimes aggravates the damage of nerve cells and tissues, and worsens the condition (Gordon, 2003; Mantovani et al., 2013). Simply inhibiting the inflammatory response will inevitably weaken its protective effect. A more principled approach is to take advantage of the benefits of the inflammatory mediator itself, while preventing the potential toxicity due to high concentrations of the mediator, and maintaining a good balance between its protective and detrimental effects.

Microglia plays an essential role in innate immunity, homeostasis, and neurotropic support in the central nervous system (Streit, 2002). Microglia is considered to be a resident macrophage in the brain and has important physiological functions. However, it is rapidly activated as a consequence of brain microenvironment changes, which induce microglia differentiation into M1 and M2 cell types (Nimmerjahn et al., 2005; Colton, 2009; Hu et al., 2015). M1 microglia is an activated form responsible of releasing large amounts of inflammatory and toxic factors with potential detrimental effects to central nervous system cells and tissues (Tang and Le, 2016; Xiong et al., 2016). M2 microglia is an alternative activation type, releasing a pool of modulatory factors including brain-derived neurotrophic factor, vascular endothelial growth factor and anti-inflammatory mediators, and promoting nerve tissue repair and nerve regeneration (Beyer et al., 2000; Jin et al., 2014; Tang and Le, 2016; Kanazawa et al., 2017; Ramirez et al., 2017). Therefore, it is of great significance to regulate the differentiation of microglia and counteract inflammatory damage.

In the central nervous system, the Notch signaling pathway is involved in dynamic changes at the cellular level which reflect into the regulation of the nervous system, which in turn plays an important role in the activation and differentiation of microglia (Grandbarbe et al., 2007). The Notch signaling pathway is mainly composed of receptors, ligand expressed on adjacent cell membranes, intracellular transcription factors, regulatory molecules and downstream effector molecules (Foldi et al., 2010). And γ-secretase catalysis is the key enzyme in the activation of Notch pathway. After the interaction between Notch receptor and ligand, the intracellular domain (notch intra-cellular domain) NICD was released into the cytoplasm and transferred into the nucleus under the catalysis of γ-secretase, which promoted the production of transcriptional activator and induced the expression downstream target genes in the Notch pathway, including hairy enhancer of split (Hes)1, Hes5, nuclear factor kappa-light-chain-enhancer of activated B cells (NF-κB), etc. (Oswald et al., 1998; Iso et al., 2003; Zhang et al., 2018). A growing number of studies have shown that the Notch signaling pathway is closely related to microglial activation and differentiation and might play a role in central nervous system diseases (Wei et al., 2011). The Notch pathway may represent a critical therapeutic target for regulating the activation and differentiation of microglia and inflammatory response. It is vital to identify drugs that regulate the activation of differentiation of Notch pathway and microglia.

Lipoxin is an endogenous anti-inflammatory lipid medium. It is released only in small amount under physiological conditions, but its synthesis is significantly increased under pathological conditions in response to inflammatory stimuli. It acts as a modulator of the inflammatory process, exerting anti-inflammatory and pro-inflammatory effects (Lee, 1995; Takano et al., 1997; Guo et al., 2016). Synthetic lipoxins such as lipoxin A4 (LXA4) possess desirable anti-inflammatory properties as shown in experimental studies of respiratory inflammation, intestinal inflammation and nephritis (Jin et al., 2007; Levy et al., 2007; Wu et al., 2007, 2009; Kure et al., 2010). Recent studies revealed that lipoxin has a protective role in central nervous system diseases such as cerebral infarction (Ye et al., 2010; Martin et al., 2014; Guo et al., 2016; Vital et al., 2016). There are very few studies investigating the interaction between LXA4 and Notch signaling pathways. Currently, only one study on transforming growth factor beta-1 (TGF-β1)-induced renal fibrosis found that LXA4 attenuated the expression of the Notch ligand Jagged1 (JAG1) and downstream molecule Hes1. Unfortunately, no further research has been carried on this important topic (Brennan et al., 2013). To date, there is no study exploring the regulatory role of LXA4 on the activation and differentiation of microglia induced by lipopolysaccharide (LPS) stimulation, nor the LXA4-mediated activation and differentiation of microglia through the Notch signaling pathway.

The purpose of this study is to clarify the modulatory effect of LXA4 on the inflammatory response associated to LPS-induced microglia activation, with a focus on the regulatory role of LXA4 on the Notch signaling pathway.

## Materials and Methods

### Cell Culture and Passage

The BV2 murine microglia cell line was purchased from the Wuhan University China Culture Collection. BV2 microglia were placed in MEM/EBSS medium containing 10% fetal bovine serum (FBS) and 100 U/ml penicillin and streptomycin, and cultured at 37°C in an incubator with a 95% O_2_/5% CO_2_ atmosphere. Every 2–3 days, the cells were washed twice with phosphate-buffered solution (PBS). After adding 1–1.5 ml of 0.125% trypsin, the attached cells were allowed to detach from the surface of the cell cultures at 37°C; the trypsin was neutralized with culture medium, and the cells were transferred into a new flask containing MEM/EBSS medium (supplemented with 10% FBS and 100 U/ml penicillin and streptomycin) and placed in the incubator. When cells grew adherent and the cell body is branched, they were transferred into 24-well plates (10^5^ cells/well for enzyme-linked immunosorbent assay [ELISA], 10^4^ cells/well for immunofluorescence), 6-well plates (2.5 × 10^5^ cells/well for quantitative reverse transcription polymerase chain reaction [qRT-PCR], 5 × 10^5^ cells/well for flow cytometry), or 100-mm culture dishes (1.2 × 10^6^ cells/dish for western blotting).

### Materials

In this study, we used the following materials: LXA4 (5S,6R,15R-trihydroxy-7,9,13-trans-11-cis-eicosatetraenoic acid; Cayman); Minimum essential medium (Eagle) with 2 mM L-glutamine and Earle’s BSS (MEM-EBSS )medium (Hyclone); FBS (Biological Industries); interleukin (IL)-1β, IL-10, and TNF-α ELISA kits (Shanghai ExCell Biotechnology); mouse anti-β-tubulin monoclonal antibody, rabbit anti-mouse inducible nitric oxide synthase (iNOS) monoclonal antibody, rabbit anti-mouse arginase (Arg)1 monoclonal antibody, and rabbit anti-mouse Notch1 monoclonal antibody (Proteintech Group); rabbit anti-mouse CD32 monoclonal antibody and rabbit anti-mouse CD206 monoclonal antibody (Abcam); mouse anti-mouse Hes1 single-clone antibody (Tianjin Sungene Biotechnology); murine anti-mouse Hes5 monoclonal antibody (Zen BioScience Co); horseradish peroxidase (HRP)-labeled goat anti-mouse secondary antibody and fluorescently labeled goat anti-rabbit secondary antibody (Tianjin Sungene Biotechnology Co); HRP-labeled goat anti-rabbit secondary antibody and anti-β-tubulin (Proteintech Group); HiScript II Q RT SuperMix for qPCR (+gDNA wiper) reagent and AceQ^®^ qPCR SYBR^®^ Green Master Mix (Low ROX Premixed) kit (Vazyme Biotechnology Co); and 2-(4-Amidinophenyl)-6-indolecarbamidine dihydrochloride, DAPI dihydrochloride sealer and LPS (Sigma), DAPT (N-[N-(3,5-difluorophenacetyl)-l-alanyl]-S-phenylglycine t-butylester) (Sigma-Aldrich, München, Germany).

### Cell Processing and Experimental Grouping

Cell treatment: BV2 microglia cultured *in vitro* stimulated by LPS as an inflammation model. Before each experiment, the cells were cultured in serum-free culture for 12 h, and LXA4 and Notch signaling pathway-specific blocker γ were administered to different groups. Pretreatment with the γ-secretase inhibitor DAPT. The concentration chosen for LPS is 200 ng/ml (Pan et al., 2016). The concentration selected for LXA4: our previous study compared the anti-inflammatory effects of 1, 10 and 100 nmol/l. It was found that the anti-inflammatory effect of 100 nmol/l was the best (Wu et al., 2011). Therefore, the study used LXA4. The concentration is 100 nmol/l. The concentration selected for DAPT is 10 μM (Wu et al., 2018).

Experimental grouping:

Part I LXA4 regulates the activation and differentiation of microglia (Results 3.1–3.2).

Control group: cells cultured in serum-free medium containing 0.035% ethanol.
LXA4 group: cells cultured in serum-free medium containing 100 nmol/l LXA4.
Lipopolysaccharide group: cells pretreated with serum-free medium containing 0.035% ethanol for 30 min, after which LPS was added to a final concentration of 200 ng/ml.
LXA4 group + LPS group: cells pretreated with serum-free medium containing 100 nmol/l LXA4 for 30 min, after which LPS was added to a final concentration of 200 ng/ml.

Part II Study on the regulation of Notch signaling pathway by LXA4 (Results 3.3).

1. LXA4 inhibits the expression of molecules downstream of the Notch signaling pathway.
  Grouped with the first part
2. LXA4 regulates Notch signaling pathway.

Control group: cells cultured in serum-free medium containing 0.035% ethanol.
LPS group: cells pretreated with serum-free medium containing 0.035% ethanol for 30 min, after which LPS was added to a final concentration of 200 ng/ml.
DAPT+LPS group: cells were pretreated with serum-free medium containing 10 μmol/l DAPT for 1 h, after which LPS was added to a final concentration of 200 ng/ml.
LXA4+LPS group: cells pretreated with serum-free medium containing 100 nmol/l LXA4 for 30 min, after which LPS was added to a final concentration of 200 ng/ml.
DAPT+LXA4+LPS group: after pretreatment with DAPT with a final concentration of 10 μM for 1 h, 100 nmol/l LXA4 was added for 30 min, and then added to a final concentration of 200 ng/ml LPS.

### ELISA for IL-1β, IL-10, and TNF-α

The concentrations of IL-1β, IL-10, and TNF-α in cell supernatants were determined by ELISA, according to the ELISA kit manufacturer’s protocol (Shanghai ExCell Biotechnology). BV2 microglia cultured on 24-well plates were treated with LPS for 6 h. Next, the cell supernatants were collected, and the total protein level therein contained was normalized for each sample prior to performing the ELISA measurements for IL-1β, IL-10, and TNF-α.

### Quantitative Reverse Transcription Polymerase Chain Reaction

Total RNA was extracted from BV2 microglia using TRIzol reagent (Invitrogen, Carlsbad, CA, United States) according to the reagent instructions. The concentration of RNA was measured using Nanodrop-1000 (Nanodrop Technologies, United States) and the purity was evaluated by the absorbance ratio at 260 and 280 nm, and the RNA purity was between 1.9 and 2.1. The cDNA was synthesized according to HiScript II Q RT SuperMix for qPCR (+gDNA wiper) (Nanjing Vazyme Biotech Biotechnology) reagent and stored at −20°C. The mRNA expression level was detected by real-time fluorescent quantitative PCR using the AceQ^®^ qPCR SYBR^®^ Green Master Mix (Low ROX Premixed) kit. The expression level of the gene of interest was normalized using GAPDH (glyceraldehyde triphosphate dehydrogenase), the CT value represents a real-time fluorescent quantitative PCR value, and the 2^−ΔΔCT^ method was used to calculate relative change analysis data of gene expression. No treatment affected the expression of GAPDH mRNA. The primer sequences used are given in the Table 1 below.

**Table 1:** Primers used for qRT-PCR.

Primer name	Sequence (5′–3′)
GAPDH	F-GGGTGTGAACCACGAGAAAT
	R-CCTTCCACAATGCCAAAGTT
Arg1	F-GACCTGGCCTTTGTTGATGT
	R-CCATTCTTCTGGACCTCTGC
iNOS	F-ACGAGACGGATAGGCAGAGA
	R-CACATGCAAGGAAGGGAACT
CD206	F-GGGACTCTGGATTGGACTCA
	R-GCTCTTTCCAGGCTCTGATG
CD32	F-GCTCAAGGAAGACACGGTGA
	R-GTGTAGCTGGCTTGGACCTG
TNF-α	F-CCGATGGGTTGTACCTTGTC
	R-AGATAGCAAATCGGCTGACG
IL-1β	F-GCTGCTTCCAAACCTTTGAC
	R-AGCTTCTCCACAGCCACAAT
IL-10	F-CCAGTTTTACCTGGTAGAAGTGATG
	R-TGTCTAGGTCCTGGAGTCCAGCAGACTCAA
Notch1	F-GCCTTCGTGCTCCTGTTCTT
	R-CTTCTTGCTGGCCTCTGACA
Hes1	F-TCATGGAGAAGAGGCGAAGG
	R-CGGAGGTGCTTCACAGTCATT
Hes5	F-AGGCCGACATCCTGGAGAT
	R-TCGCTGTAGTCCTGGTGCAG

### Western Blot Analysis

BV2 microglia cells were treated with LPS for 4 and 8 h, and the cell culture medium was discarded and washed three times with sterile phosphate buffered saline. A 100:1 mixture of 100 μl of ice-cold cell lysis buffer and protease inhibitor (PMSF) was added to the cell culture, then cells were incubated on ice for 30 min, and the lysate was clarified by spinning for 10 min at 4°C (12,000 rpm), leaving the supernatant for later use. Protein quantification was performed using the micro bicinchoninic acid method. A 5× sodium dodecyl sulfate loading buffer was added, before incubating at 99°C for 5 min. Then the samples were loaded at 15 μg protein/lane on 6 or 12% acrylamide gels and subjected to sodium dodecyl sulphate polyacrylamide gel electrophoresis for about 1.5 h at 80 mV (stacking gel) and 120 mV (resolving gel). Proteins were then transferred to a polyvinylidene fluoride membrane and blocking was made for 2 h in a 5% non-fat dry milk in Tris base/Tween-buffered saline (TBST). The molecular weights of the iNOS, Notch1, arginase (Arg)1, Hes1, Hes5, and β-tubulin proteins are 131, 120, 35/38, 35, 18, and 55 kDa, respectively. Samples were incubated at 4°C overnight with Rabbit anti-iNOS (1:500), Notch1 (1:500) and mouse anti-Arg1 (1:300), Hes1 (1:500), Hes5 (1:300), β-tubulin (1:1,000) primary antibodies. After washing three times with TBST, the samples were incubated for 1 h at room temperature with a secondary antibody of the IgG family, conjugated with HRP. Enhancement of the antibody reaction using an hypersensitive chemiluminescent (ECL) reagent (Beyotime Biotechnology) allowed for visualizing the protein. Protein bands were quantified using the ImageJ software and the band intensity was normalized to the band intensity of β-tubulin.

### Immunofluorescence

To detect the expression of BV2 M1 and M2 microglia biomarkers, we first treated the cells with LPS. After treatment, the cells were washed 3 × 5 min with PBS (pH 7.4), and 4% paraformaldehyde was added for 30 min at room temperature, after which the cells were again washed 3 × 5 min with PBS. The membranes were disrupted with 0.5% Triton X-100 (PBS configuration) for 5 min. After washing three times with PBS for 5 min each, we added PBS containing 2% bovine serum albumin and 10% goat serum, and the plates were sealed at 37°C for 45 min. The following antibodies were added overnight at 4°C: rabbit anti-iNOS (1:200), rabbit anti-CD32 (1:200), rabbit anti-Arg1 (1:100), and rabbit anti-CD206 (1:100). After washing 3 × 5 min with PBS, we added a fluorescently labeled goat anti-rabbit IgG secondary antibody (1:500) for 1 h at room temperature. The cells were again washed 3 × 5 min with PBS, and counterstained in the dark with a mixture of 1 ml DAPI (1 mg/ml) + 1 ml H_2_O. The cells were incubated at 37°C for 10 min, washed 3 × 5 min with PBS, observed under an Inverted fluorescence microscope (IX71 Japan OLYMPUS Corp.), and photographed.

### Flow Cytometry

BV2 microglia were seeded at 5 × 10^5^/well and treated according to their experimental group. After treatment, the culture supernatant was discarded, and cells were washed gently with 2 ml of PBS. Then, the PBS was aspirated and the adherent cells were digested with 1 ml of trypsin; 1 ml of MEM/EBSS medium was used to stop the digestion. The cells were centrifuged (1,000 rpm, 5 min, 4°C), washed twice with PBS, and resuspended at 1 × 10^5^ cells/ml. To label the cells, we added PE/Cy5 anti-mouse CD16/CD32 monoclonal antibody (≤0.25 μg/10^6^ cells, 100 μl) and Alexa Fluor^®^ 488-labeled anti-mannose receptor antibody (1:500) for 20 min at room temperature in the dark, washed the cells twice with PBS, and resuspended them in 300 μl PBS. The cell surface expression of CD16/CD32 and CD206 was detected using a FACS Calibur flow cytometer (BD Company), and the data were analyzed using FlowJo software.

### Statistical Analysis

All data are expressed as mean ± SD. Statistical analysis was performed using SPSS 21.0 statistical software. One-way analysis of variance (ANOVA) was used for comparison between groups, and pairwise comparison between sample means was performed using the Bonferroni method. Statistical significance was set at *p* < 0.05.

## Results

### LXA4 Affects the Expression of Interleukin (IL)-1β, IL-10, and TNF-α in LPS-Treated BV2 Microglia

**FIGURE 1:**
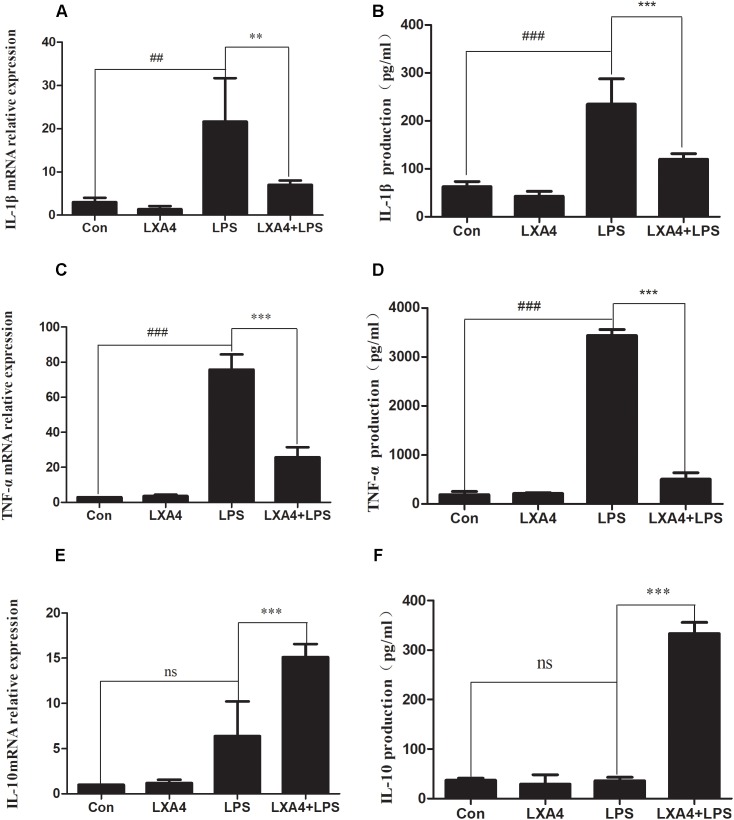
LPS-induced microglia activation and release of inflammatory factors; After 6 h of LPS stimulating **(A,C,E)**: the mRNA expression levels of IL-1β, TNF-α were measured by real-time fluorescent quantitative PCR; 8 h after LPS treatment, **(B,D,F)** The concentration of IL-1β, TNF-α and IL-10 in the supernatant of the cells was detected by enzyme-linked immunosorbent assay (ELISA). LPS induces microglia activation, proinflammatory cytokines IL-1β, TNF-α and anti-inflammatory factor IL-10 release. With LXA4 intervention, IL-1β, TNF-α expression decreased, IL-10 expression increased significantly, ^##,^∗∗^^*p* < 0.01; ^###,^∗∗∗^^*p* < 0.001; n.s., no significance.

The expression of IL-1β, TNF-α and IL-10 mRNA was determined by qRT-PCR 6 h after the corresponding treatment. LPS induced a significant increase in IL-1β and TNF-α mRNA in BV2 microglia as compared to the control group (*p* < 0.05) (Figure 1A,C). LXA4 pretreatment reduced LPS-induced IL-1β and TNF-α mRNA levels (Figure 1A,C), while IL-10 mRNA expression increased significantly (*p* < 0.05) (Figure 1E). That is, LXA4 inhibited the expression of IL-1β and TNF-α mRNA in BV2 microglia induced by LPS, and upregulated the mRNA expression of IL-10.

Eight hours after the corresponding treatment, the ELISA method was used to detect the protein expression levels of IL-1β, TNF-α and IL-10. As compared to the control group, LPS induced a significant increase in IL-1β and TNF-α protein levels in BV2 microglial culture supernatants, and the difference was statistically significant (*p* < 0.05) (Figure 1B,D). LXA4 pretreatment inhibited the production of IL-1β and TNF-α induced by LPS (*p* < 0.05) (Figure 1B,D), while the protein level of IL-10 was significantly higher than that of the LPS group (*p* < 0.05) (Figure 1F). That is, LXA4 inhibited the expression of IL-1β and TNF-α mRNA induced by LPS in BV2 microglia, and upregulated the protein expression of IL-10.

Therefore, LXA4 inhibited the genes and protein expression of M-1 microglia-associated inflammatory factors IL-1β and TNF-α, and upregulated the expression of IL-10, an inflammatory factor associated with M2 microglia.

### LXA4 Affects LPS-Induced BV2 Microglial Morphological Changes and Induces the Conversion of (M1) Microglia to (M2) Microglia

**FIGURE 2:**
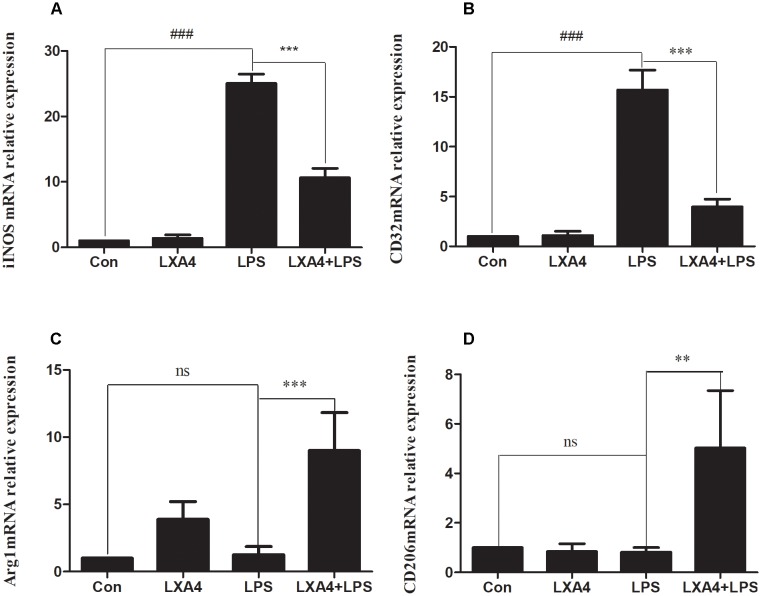
Quantitative RT-PCR analysis of iNOS, CD32, Arg1, CD206 mRNA levels in the BV2microglia 6 h after LPS treatment. **(A,B)** Quantitative RT-PCR analysis of iNOS, CD32 mRNA levels. **(C,D)** Quantitative RT-PCR analysis of Arg1, CD206 mRNA levels. LPS upregluated M1 microglia biomarkers iNOS, CD32. With LXA4 intervention, iNOS, CD32 expression decreased, M2 microglia biomarkers Arg1, CD206 expression increased significantly. ^##,∗∗^*p* < 0.01; ^###,∗∗∗^*p* < 0.001; n.s., no significance.

The expression of iNOS, cluster of differentiation (CD)32, Arg1 and CD206 mRNA was detected via qRT-PCR 6 h after the corresponding treatment. As compared to the control group, the relative expression of iNOS and CD32 mRNA in the LPS group was significantly increased (*p* < 0.05) (Figure 2A,B). However, the relative expression of mRNA of the M2 markers Arg1 and CD206 did not change significantly (*p* > 0.05). After LXA4 pretreatment, the expression of iNOS and CD32 was both significantly decreased (*p* < 0.05) (Figure 2A,B), and the expression of Arg1 and CD206 was both significantly increased (*p* < 0.05) (Figure 2C,D). Therefore, LXA4 inhibited the expression of the M1 markers iNOS and CD32 at the transcriptional level and upregulated the expression of the M2 markers Arg1 and CD206.

Each group was tested 8 h after the corresponding treatment. The levels of iNOS, Arg1, CD32 and CD206 proteins were determined by Western blot, cellular immunofluorescence and flow cytometry.

**FIGURE 3:**
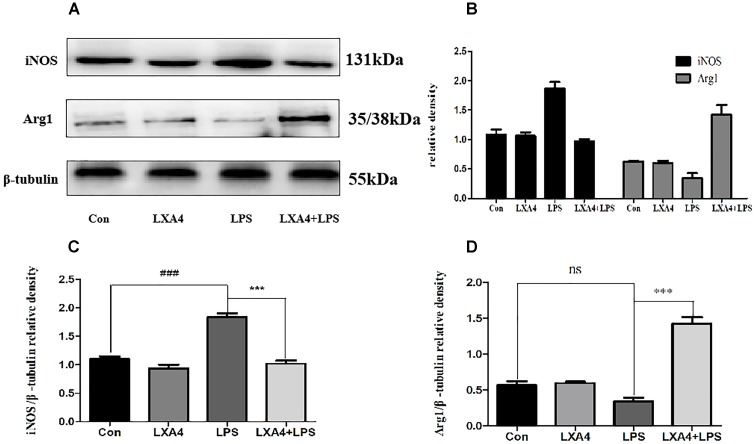
Western blot analysis of the effect of LXA4 on iNOS and Arg1. **(A–C)** Western blot analysis of the protein level of iNOS in microglia. **(A,B,D)** Western blot analysis of the protein level of Arg1 in microglia, LPS upregluated M1 microglia biomarkers iNOS. With LXA4 intervention, iNOS expression decreased, M2 microglia biomarkers Arg1 expression increased significantly. ^###,∗∗∗^*p* < 0.001; n.s., no significance.

Western blot analysis showed that the expression of iNOS protein in LPS group was significantly increased as compared to the control group (*p* < 0.05) (Figure 3A–C), while the expression of Arg1 protein did not significantly increase (*p* > 0.05). As compared to the LPS group, the expression of iNOS protein was inhibited and the expression of Arg1 protein was increased in the LXA4 pretreatment group (*p* < 0.05) (Figure 3A–D).

**FIGURE 4:**
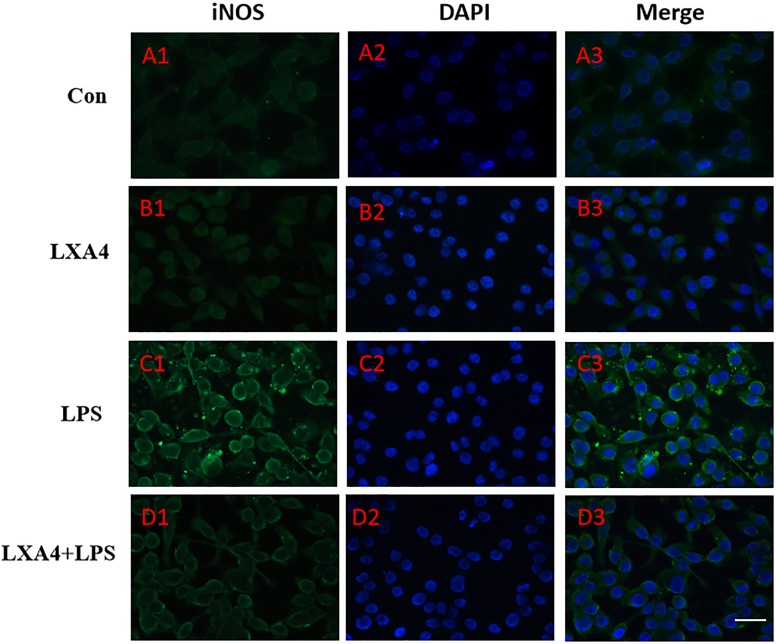
Immunofluorescence images showing the BV2 microglia after LPS stimulates which were labeled with iNOS antibody, with LXA4, the expression of iNOS is decreased. Green fluorescence indicated iNOS positive cells, while blue fluorescence indicated DAPI-labeled nuclei. Scale bar: 20 μm.

**FIGURE 5:**
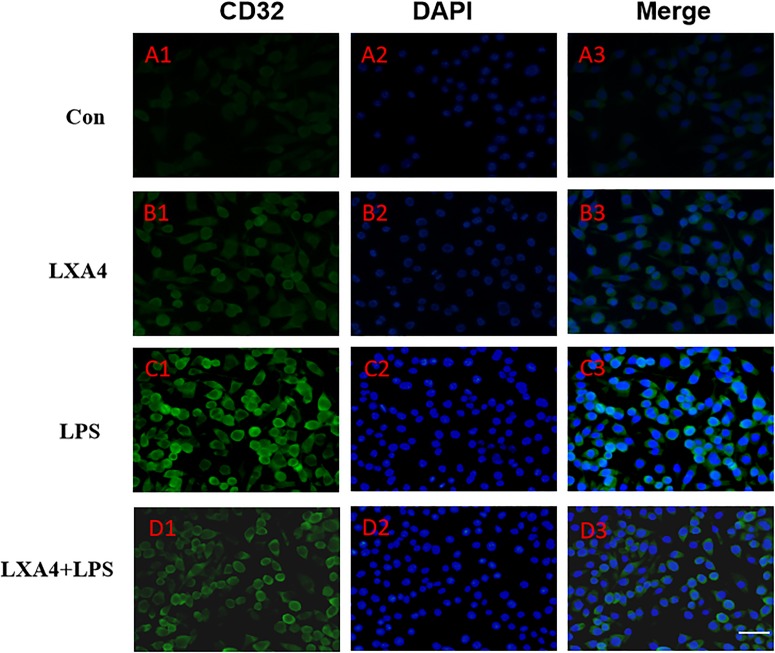
Immunofluorescence images showing the BV2 microglia after LPS stimulates which were labeled with CD32 antibody, with LXA4, the expression of iNOS is decreased. Green fluorescence indicated CD32 positive cells, while blue fluorescence indicated DAPI-labeled nuclei. Scale bar: 20 μm.

**FIGURE 6:**
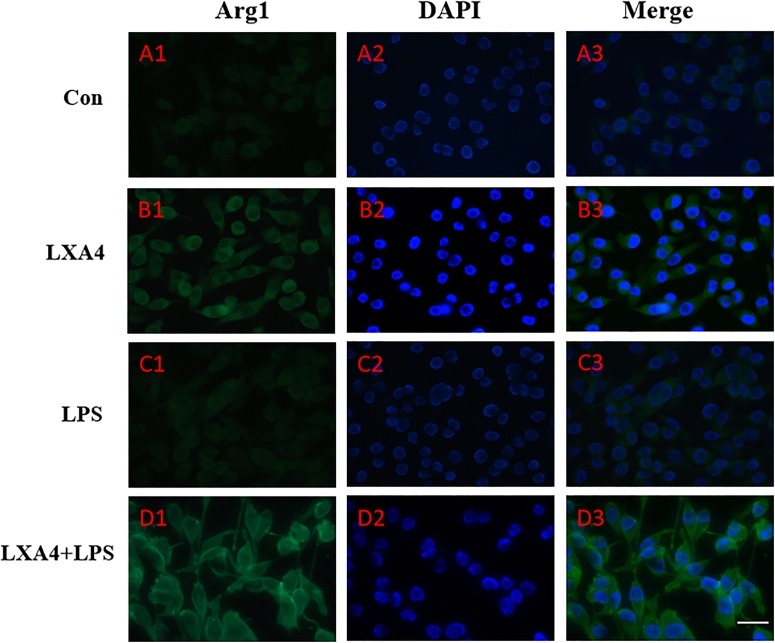
Immunofluorescence images showing the BV2 microglia after LPS stimulates which were not labeled with Arg1 antibody, with LXA4, the expression of Arg1 is expressed at high levels. Green fluorescence indicated Arg1 positive cells, while blue fluorescence indicated DAPI-labeled nuclei. Scale bar: 20 μm.

**FIGURE 7:**
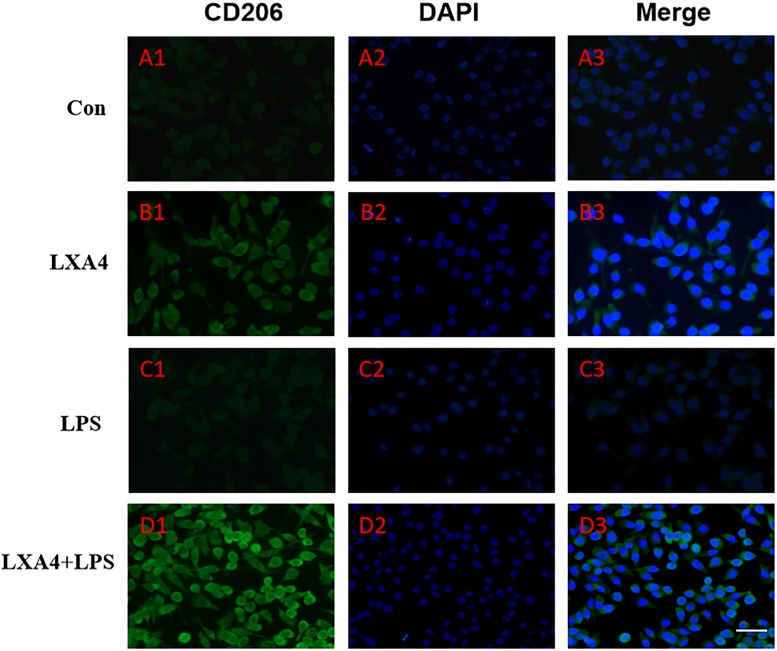
Immunofluorescence images showing the BV2 microglia after LPS stimulates which were not labeled with CD206 antibody, with LXA4, the expression of Arg1 is expressed at high level. Green fluorescence indicated CD206 positive cells, while blue fluorescence indicated DAPI-labeled nuclei. Scale bar: 20 μm.

Cellular immunofluorescence showed that BV2 microglia cells were activated in response to LPS treatment, the cell body became larger and rounder, the protrusion decreased and became thicker, and the morphology appeared as amoeba-like. LXA4 pretreatment weakened the response to LPS in terms of morphological changes (Figure 4–7). As compared to the control group, the expression of iNOS and CD32 after LPS treatment increased (*p* < 0.05) (Figure 4, 5). As compared to the LPS group, the expression of iNOS and CD32 in the LXA4 pretreatment group was higher than that in the LPS group. The expression of Arg1 and CD206 significantly decreased in the LPS group (*p* < 0.05) (Figure 6, 7).

**FIGURE 8:**
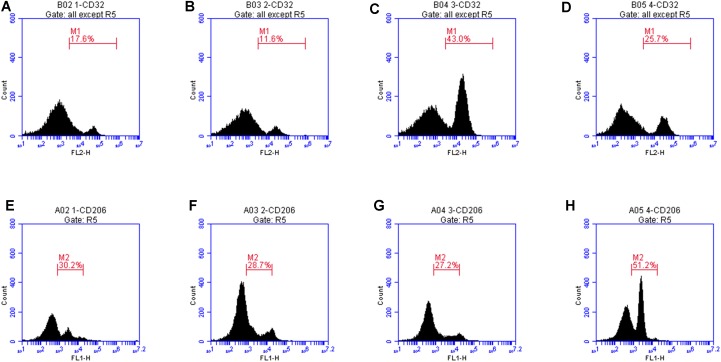
Protein expression levels of CD32 and CD206 were measured by flow cytometry. **(A)** The mean fluorescence intensity of CD32 was only 17.6. **(B)** Treatment with LXA4 alone, the mean fluorescence intensity was 11.6. **(C)** In the LPS group, the average fluorescence intensity increased to 41.3. **(D)** In the LPS group pretreated with LXA4, the mean fluorescence intensity was only 25.7. **(E)** In the control group, the mean fluorescence intensity of CD206 was only 30.2, **(F)** In the group treated with LXA4 alone, the mean fluorescence intensity of CD206 was 28.7. **(G)** In the LPS group, the mean fluorescence intensity of CD206 was 27.2. **(H)** In the LPS group pretreated with LXA4, the average fluorescence intensity significantly increased to 51.2.

CD32 is a M1 microglia surface marker molecule and can be detected by flow cytometry. As shown in Figure 8, we observed positive expression of CD32. The mean fluorescence intensity was only 17.6, indicating a low expression level. Treatment with LXA4 alone induced positive expression of CD32 and the mean fluorescence intensity was 11.6 (Figure 8B). In the LPS group, the average fluorescence intensity increased to 41.3, indicating that CD32 positive expression was significantly enhanced. In the LPS group pretreated with LXA4, positive expression was observed, but the mean fluorescence intensity was only 25.7. On the other side, CD206 is a M2 type microglia surface marker molecule. As shown in Figure 8E, the control group showed positive expression of CD206. However, the mean fluorescence intensity was only 30.2, indicating a low expression level. In the group treated with LXA4 alone, a positive expression was observed, with a mean fluorescence intensity value of 28.7, indicating a low expression level. In the LPS group, the mean fluorescence intensity increased to 27.2, showing low expression level. In the LPS group pretreated with LXA4, the average fluorescence intensity significantly increased to 51.2 (*p* < 0.05). That is, LXA4 inhibits the expression of CD32 and upregulates the expression of CD206.

Western blot, cellular immunofluorescence and flow cytometry were concordant in indicating that LXA4 inhibited the expression of M1 type biomarkers iNOS and CD32 at the protein expression and transcription levels, and upregulated the M2 type biomarkers Arg1 and CD206. In other words, LXA4 promoted the shift of M1 to M2 microglia.

### Mechanism of LXA4 Regulation of Notch Signaling Pathway

#### Preliminary Observations of the Effect of LXA4 on the Expression of Downstream Effector Molecules in the Notch Signaling Pathway

**FIGURE 9:**
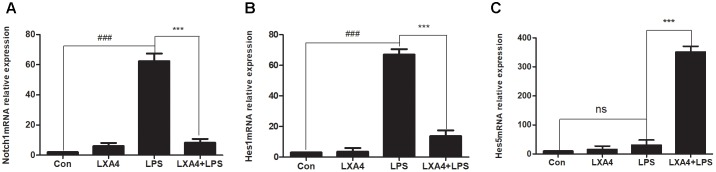
Quantitative RT-PCR analysis of Notch1, Hes1 and Hes5 mRNA levels in the BV2 microglia. **(A)** Quantitative RT-PCR analysis of Notch1 mRNA levels. **(B)** Quantitative RT-PCR analysis of Hes1 mRNA levels. **(C)** Quantitative RT-PCR analysis of Hes5 mRNA levels.LPS upregluated M1 microglia related signal molecule Notch1, Hes1. With LXA4 intervention, Notch1, Hes1 expression decreased, M2 microglia related signal molecule Hes5 expression increased significantly. ^###,∗∗∗^*p* < 0.001; n.s., no significance.

The relative expression levels of Notch1, Hes1 and Hes5 mRNA were determined by qRT-PCR 3 h after the corresponding treatments were administered. As compared to the control group, the expression of Notch1 and Hes1 mRNA in the LPS group was significantly different (*p* < 0.05) (Figure 9A,B), while the relative expression of Hes5 mRNA was not significantly increased (*p* > 0.05) (Figure 9C). As compared to the LPS group, the expression of Notch1 and Hes1 mRNA in the LXA4 pretreatment group was decreased, and the expression of Hes5 mRNA was significantly increased (*p* < 0.05) (Figure 9A–C).

**FIGURE 10:**
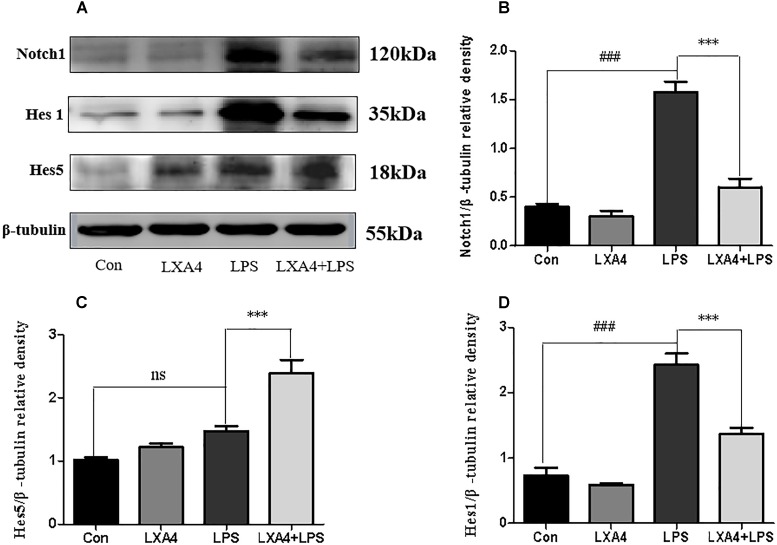
Western blot analysis of the effect of LXA4 on Notch1, Hes1 and Hes5. **(A,B,D)** Western blot analysis of the protein level of Notch1, Hes1 in microglia. **(A,C)** Western blot analysis of the protein level of Hes5 in microglia, LPS upregluated M1 microglia related signal molecule Notch1, Hes1. With LXA4 intervention, Notch1, Hes1 expression decreased, M2 microglia related signal molecule Hes5 expression increased significantly. ^###,∗∗∗^*p* < 0.001; n.s., no significance.

Each group was performed for 4 h with corresponding treatments and the expression levels of Notch1, Hes1, and Hes5 proteins were determined through Western blot. The expression of Notch1 and Hes1 protein in the LPS group was significantly higher than in the control group (*p* < 0.05), but the expression of Hes5 was not significantly increased. The difference was not statistically significant (*p* < 0.05). As compared to the LPS group, the expression of Notch1 and Hes1 protein in the LXA4 pretreatment group was decreased, and the protein expression of Hes5 was significantly increased (*p* < 0.05) (Figure 10).

Therefore, LXA4 affected the expression of downstream effector molecules within the Notch signaling pathway at the level of genes and protein; inhibited the expression of Notch1 and Hes1, and upregulated the expression of Hes5.

#### LXA4 Regulation of the Notch Signaling Pathway

##### LXA4 regulation on downstream effector molecules of the Notch signaling pathway

**FIGURE 11:**
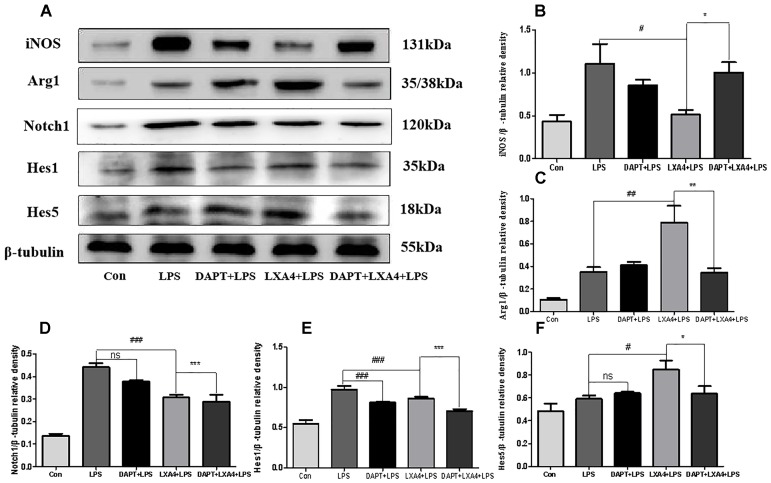
Western blot analysis of the Notch1, Hes1, Hes5, iNOS and Arg1 effect after DAPT pretreatment. **(A–C)** Western blot analysis of the protein level of iNOS, Arg1 in microglia. **(A,D–F)** Western blot analysis of the protein level of Notch1, Hes1, Hes5 in microglia. ^#,^∗^^*p* < 0.05; ^##,^∗∗^^*p* < 0.01; ^###,^∗∗∗^^*p* < 0.001; n.s., no significance.

Each group was performed for 6 h with corresponding treatments and the expression levels of Notch1, Hes1, and Hes5 proteins were determined through Western blot. The levels of Notch1 and Hes1 protein in the LPS group were significantly higher than in the control group (*p* < 0.05), and there was no significant Hes5 upregulation (*p* > 0.05) (Figure 11A,D–F). In the DAPT-pretreatment group as compared to the LPS group, the Hes1 protein level was significantly decreased (*p* < 0.05) (Figure 11A,E), the Notch1 protein decreased slightly, and the Hes5 protein increased, but the change was not statistically significant (*p* > 0.05). In the LXA4 pretreatment group, the levels of Notch1 and Hes1 protein significantly decreased (*p* < 0.05) and the Hes5 protein was significantly upregulated (*p* < 0.05) (Figure 11A,D–F). After combined DAPT/LXA4 pretreatment, Notch1, Hes1 and Hes5 group were significantly decreased (*p* < 0.05). There was no change between Hes1 and Hes5 as compared to the control group.

Therefore, LXA4 downregulated the expression of Notch1 and the downstream effector Hes1 in M1 microglia differentiation (Wang et al., 2010; Liu et al., 2012; Wu et al., 2018), and upregulated the downstream effector Hes5 associated with the M2 differentiation (Liu et al., 2012), promoting the transformation of M1 to M2 microglia. However, after blocking the Notch signaling pathway with the γ-secretase inhibitor DAPT, the LXA4 regulation on the downstream effector molecules Hes1 and Hes5 of the Notch signaling pathway was abolished, indicating that LXA4 regulates the differentiation of microglia through the Notch signaling pathway.

##### Effect of LXA4 on microglia differentiation

Each group was performed for 6 h with corresponding treatments and the expression levels of microglia M1 biomarker iNOS and M2 biomarker Arg1 proteins were determined through Western blot. The expression of the iNOS protein increased in the LPS group as compared to the control group (Figure 11A–C). After treatment with DAPT and LXA4, the level of the iNOS protein was decreased, while after LXA4 pretreatment, the level of Arg1 protein was upregulated (Figure 11A–C). That is, LXA4 promotes the conversion of M1–M2 phenotype; the action of LXA4 can be abolished by the Notch signaling pathway blocker DAPT. The figure shows that the expression of the iNOS protein in the DAPT+LXA4+LPS group was increased, and the expression of Arg-1 was not, confirming that LXA4 regulates the differentiation of microglia through the Notch signaling pathway.

##### Effect of LXA4 on Notch signaling pathway and microglial differentiation on expression of inflammatory mediators

**FIGURE 12:**
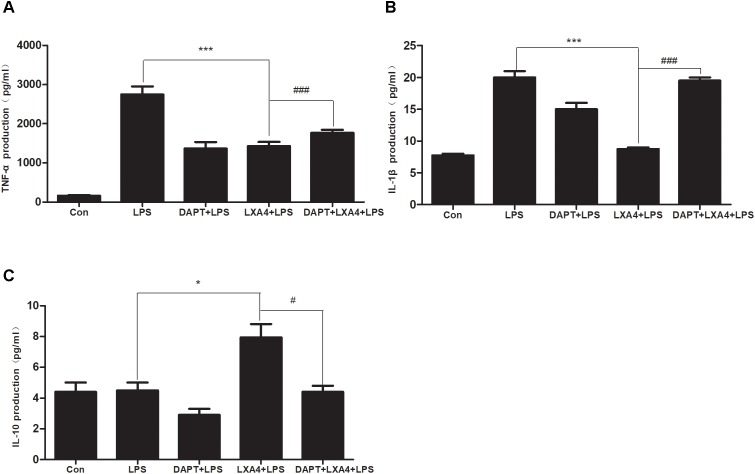
LPS-induced microglia activation and release of inflammatory factors; After 6 h of LPS stimulating **(A–C)**: the protein expression levels of IL-1β, TNF-α, IL-10 were measured by enzyme-linked immunosorbent assay (ELISA). ^#,^∗^^*p* < 0.05; ^##,^∗∗^^*p* < 0.01; ^###,^∗∗∗^^*p* < 0.001; n.s., no significance.

Each group was performed for 6 h with corresponding treatments and the ELISA method was used to detect the expression levels of M1 microglia-associated inflammatory cytokines IL-1β and TNF-α and M2 microglia-associated inflammatory factor IL-10. As compared to the control group, the expression of IL-1β and TNF-α protein in the LPS group increased (Figure 12A,B); as compared to the LPS group, the expression of IL-1β and TNF-α in the DAPT+LPS group was significantly decreased (*p* < 0.05) (Figure 12A,B). The expression of IL-10 was upregulated in the LXA4+LPS group (Figure 12A,B), while IL-1β and TNF-α significant decreased (Figure 12C), suggesting that LXA4 promoted the conversion of M1 to the M2 phenotype, and this effect was abolished by the Notch signaling pathway blocker DAPT. As shown in Figure 12C, the inflammatory factors IL-1β and TNF-α are upregulated in the DAPT+LXA4+LPS group, while IL-10 showed no significant upregulation, confirming that the LXA4 modulates the expression of inflammatory cytokines by regulating the differentiation of microglia through the Notch signaling pathway.

## Discussion

An increasing number of studies reveal that inflammatory reactions exert a significant influence on the occurrence and development of central nervous system conditions such as ischemic stroke, Alzheimer’s disease, Parkinson’s disease, and multiple sclerosis, at multiple stages of the disease process. Inflammation aggravates the damage to nerve cells and tissues, worsening the pathological condition (Lucas et al., 2006; Xanthos and Sandkuhler, 2014; Meng et al., 2016).

The role of microglia in the neuroinflammatory response is important (Streit, 2002; Ramirez et al., 2017). Microglia cells are related to mononuclear/macrophage cell lines as for morphology, immunophenotype, and biological function, and are considered to be resident macrophages in the brain (Streit, 2000). Under physiological conditions, microglia plays an important role in the development, structural formation and functional regulation of the nervous system (Kim and de Vellis, 2005; Gomez-Nicola and Perry, 2015; Wolf et al., 2017). After exogenous stimulation or microenvironment changes in the brain, microglia is rapidly activated, and a series of changes occur in cell morphology, immunophenotype and function. Activated microglia mainly differentiate into M1 and M2 types (Kloss et al., 2001; Hu et al., 2014). M1 type is a classical activated microglia, and the corresponding biological molecular markers include iNOS and CD32. M1 microglia releases a large number of inflammatory factors such as TNF-α and IL-1β, causing damage to central nervous system cells and tissues. Increased expression of iNOS produces a large amount of NO in the brain, and high-load NO can exert toxic effects through various mechanisms such as mitochondrial damage, peroxidation, activation and inhibition of various signaling pathways, and DNA damage (Pacher et al., 2007; Martinez et al., 2009). The M2 type is an alternative activation type, and the corresponding biological molecular markers include Arg1 and CD206. M2 microglia can release chemokines, induce resting microglia to focus on the lesion, phagocytose toxic molecules and cell debris, and release brain-derived neurotrophic factor, vascular endothelial growth factor and the anti-inflammatory mediator IL-10 which inhibits immune inflammation, promotes inflammation regression, as well as nerve tissue repair and nerve regeneration (Jin et al., 2014; Beyer et al., 2015; Tang and Le, 2016; Kanazawa et al., 2017; Ramirez et al., 2017). M2 microglia releases Arg1, which competes with iNOS for arginine substrate, downregulates NO production, reduces tissue damage, and participates in tissue damage repair (Yang et al., 2016). Regulating the differentiation of microglia, and avoiding harm, is of great significance for the improvement of the prognosis of various inflammatory related nervous system diseases.

Activation and differentiation of microglia involves multiple signaling pathways, such as the Notch signaling pathway, NF-κB, tyrosine protein kinase (JAK) signal transduction/transcriptional activator (STAT), peroxisome proliferator-activator receptor-γ (PPAR-γ) and cAMP response element binding protein (CREB) (Grandbarbe et al., 2007; Xu et al., 2015; Ghosh et al., 2016; Chen J. et al., 2017; Qin et al., 2017; Wei et al., 2017; Liu et al., 2018). The Notch signaling pathway is highly conserved and participates in almost all physiological and pathological processes such as differentiation, proliferation and apoptosis of all cellular types. It can precisely regulate cell differentiation by translocating signals directly from adjacent cells to the cell nucleus to activate transcription factors, affecting embryonic development and the homeostasis of adult tissues and organs (Gazave et al., 2009; Oya et al., 2009; Ables et al., 2011; Andersson et al., 2011). In the central nervous system, the Notch signaling pathway is actively involved in dynamic changes at all scales, from cellular structure to nervous system function, inhibiting neuronal differentiation and promoting differentiation of glial subtypes (Tanigaki et al., 2001), especially during microglia activation. Moreover, it plays a very important role in differentiation (Grandbarbe et al., 2007; Yuan et al., 2015). The Notch signaling pathway is mainly composed of receptors, ligands expressed on adjacent cell membranes, intracellular transcription factors, regulatory molecules, and downstream effector molecules (Shang et al., 2016). After the interaction between the Notch receptor and the ligand, the NICD is released into the cytosol, thanks to the cleavage operated by the key enzyme γ-secretase which directly affected the activation of Notch pathway, and transferred to the nucleus. The activated NICD promotes the production of transcriptional activators, thereby inducing the expression of downstream migratory molecules including Hes1, Hes5 and NF-κB. Some studies have shown that elevated levels of Notch1 receptor and downstream effector Hes1 are associated to microglia differentiation into the M1 type (Wang et al., 2010; Liu et al., 2012; Wu et al., 2018), while Hes5 is involved in M2 differentiation (Liu et al., 2012). Downstream target genes of the Notch pathway include Hes1, Hes5, NF-κB, Cyclin D1, and C-myc. The Notch pathway may act as a cascade with complex interactions with the NF-κB, Wnt, TGF/BMP, TLR and other pathways (Wei et al., 2011). The Notch pathway may be important for regulating the activation and differentiation of microglia and the inflammatory response. More and more studies show that the Notch signaling pathway and microglia activation and differentiation are involved in central nervous system diseases. The modulation of these processes is expected to be a key to the treatment of several central nervous system conditions. It is therefore imperative to identify drugs that regulate the Notch pathway and microglia activation and differentiation.

Lipoxins (LXs) are a class of arachidonic acid-derived mediators formed via lipoxygenase-catalyzed reactions, which carry anti-inflammatory and pro-inflammatory properties, and are classified according to the position and conformation of the hydroxyl groups in the molecule. LXA4 and LXB4, and their epimers 15-epi-LXA4 and 15-epi-LXB4, are synthesized only in small amount under physiological conditions. However, their levels significantly rise under various pathological conditions involving inflammatory stimuli, to act as downregulators of the inflammatory process. LXs play a role in anti-inflammatory and pro-inflammatory decline. Some synthetic lipoxins such as LXA4 have exhibited anti-inflammatory effects in experimental studies on respiratory tract infections, lung injury, peritonitis, enteritis, nephritis, gynecological inflammation, and various tumor-related inflammations (Gewirtz et al., 2002; Chen et al., 2010; Xu et al., 2012, 2014; Okano Kwangbo Department of Medical Science and Research of Okayama University Department of Otolaryngology, 2015; Borgeson et al., 2015; Qi et al., 2015; Wang Z. et al., 2015; Chen X.Q. et al., 2017; Xu et al., 2018). Experimental studies conducted in recent years have also found that lipoxin has a regulatory effect on central nervous system diseases such as cerebrovascular diseases and Alzheimer’s disease (Wu et al., 2013; Guo et al., 2016; Han et al., 2016; Kantarci et al., 2018). The LXA4 receptor is represented on the microglia surface (Cui et al., 2002; Chen et al., 2006), which means that microglia may also be a target for the LXA4 in the central nervous system. There is very little research on whether LXA4 can act on the Notch signaling pathway. Only one study found that the LXA4 attenuates TGF-β1-induced renal fibrosis by suppressing the Notch signaling pathway (Brennan et al., 2013). However, whether the LXA4 can function through the Notch signaling pathway in the central nervous system diseases has not been reported before, especially in reference to the regulation of microglia activation and differentiation. Our study clarified that the LXA4 regulates microglia activation and differentiation through the Notch signaling pathway and plays an anti-inflammatory role, providing a new therapeutic target for the treatment of inflammatory-related nervous system diseases.

In this study, the immortalized murine microglial cell line BV-2 was used to construct an inflammatory model. BV2 microglia retains many morphological features, phenotypical characterization, and functional characteristics of the microglia, and is consequently an ideal model for studying microglia. We found that after LPS stimulation of BV2 microglia, the microglia cells were activated, and the round, swelling, and thin processes of the cell body retracted from branching to an amoeba-like asset. After pretreatment with LXA4, microglia cells showed small bodies and many branches.

This investigation capitalized on a complete set of analyses, including qRT-PCR, ELISA, western blot, cell immunofluorescence, and flow cytometry. A first part of the study explored the regulatory role of the LXA4 on the activation and differentiation of the microglia. We found that LXA4 could inhibit gene and protein expression of M1 biomarkers iNOS, CD32 and M1 related inflammatory cytokines IL-1β and TNF-α. Conversely, LXA4 caused upregulation of the expression of M2 biomarkers Arg1 and CD206 and M2 microglia-associated inflammatory factor IL-10. LXA4 can regulate the switch from M1 to M2 microglia and alleviate inflammation. A second part of this study focused on the LXA4 regulation of the Notch signaling pathway. LXA4 could affect the expression of downstream effector molecules of the Notch signaling pathway at both the gene and protein levels: it inhibited the expression of Notch1 and Hes1 related to the differentiation of M1 microglia. Upregulating the expression of Hes5 in association with M2 differentiation suggests that LXA4 promotes the transformation of M1 type to M2 microglia through the Notch signaling pathway.

It was subsequently found that the specific blocker Notch signaling pathway and the regulation of Notch downstream effector molecules Hes1 and Hes5 by LXA4 were blocked after the treatment of γ-secretase inhibitor DAPT. The results suggest that LXA4 regulates the differentiation of microglia through Notch signaling pathway.

Further studies showed that LXA4 decreased the expression of M1 related biomarkers iNOS and the related inflammatory cytokines IL-1β and TNF-α. The upregulation of the expression of Arg1 and the related inflammatory factor IL-10 can also be blocked by the DAPT. Therefore, we finally confirmed that LXA4 can exert its anti-inflammatory effects by regulating the differentiation of microglia through the Notch signaling pathway.

In conclusion, LXA4 inhibited the activation of microglia induced by LPS, promoted the transformation of M1–M2, and reduced the expression of IL-1β, TNF-α, and iNOS, while enhancing the expression of the anti-inflammatory mediator IL-10 through the Notch signaling pathway. This study shows: 1. LPS stimulation induced M1 microglia activation and increased the secretion of pro-inflammatory factors; 2. The Notch1 receptor and downstream effector Hes1 increased, suggesting that microglia differentiated into M1 type; 3. We revealed that LXA4 has desirable anti-inflammatory properties, which is consistent with previous studies (Wang et al., 2011; Zhou et al., 2011). But most importantly, we proved for the first time that LXA4 can regulate the differentiation of microglia and inhibit the inflammatory response induced by LPS, especially through the Notch signaling pathway, which is the focus and bright spot of this study.

It should be highlighted that γ-secretase is a key enzyme in the activation of the Notch pathway. DAPT can inhibit the activation of the Notch pathway by inhibiting the γ-secretase. However, the expression of the Notch1 protein was not affected in theory. Therefore, the expression of Hes1 and Hes5 was mainly inhibited after DAPT administration, and the change of Notch1 was not significant. Previous studies have shown that the γ-secretase enzyme blockers affect the Notch signaling pathway and produce a series of side effects at the level of the gastrointestinal tract and hematopoietic system, as well as induce thymocyte damage (Mumm et al., 2000; Real and Ferrando, 2009; Coric et al., 2012). Therefore, γ-secretase blockers are relatively far away from clinical use. A large number of studies have confirmed that lipoxin has the ability of inhibiting the further deterioration of inflammation *in vivo* and *in vitro*, promoting the timely regression of inflammation (Wang et al., 2014; Yao et al., 2014). Lipoxin acts in local tissues after its production, and then rapidly deactivates, it does not interfere with normal physiological function, is safe, has no toxic side effects, and can be used as an anti-inflammatory and modulating substance. Based on these advantages, and on its ability to maintain a balance between the benefits of the inflammatory mediators themselves and their potential toxicity at high concentrations (Zhou et al., 2011; Zhao et al., 2013), Lipoxin is expected to be a new anti-inflammatory drug, especially in the treatment of inflammatory nervous system diseases. Previous studies have also emphasized the expression of LXA4 receptors in neurons, microglia, astrocytes, and neural stem cells in the central nervous system (Wada et al., 2006; Decker et al., 2009; Wang et al., 2011). This suggests that they may be the target of lipoxin action in the central nervous system. Future studies will focus on ascertain the interplay between lipoxin-mediated pathways and other cells, notably neurons, astrocytes, and neural stem cells, to further confirm the anti-inflammatory effect of lipoxin, reveal its anti-inflammatory mechanisms, and provide more evidence for clinical applications.

## Author Contributions

JW and D-hD participated in the design of this study and they both carried out the study and performed the statistical analysis. JW collected important background information and D-hD drafted the manuscript, and JW repeatedly modified the text structure and Details. Q-qL, X-yW, Y-yS, and L-JL participate in the original article and discussion about article writing and revision. All the authors revised and approved final version of the manuscript.

## Conflict of Interest Statement

The authors declare that the research was conducted in the absence of any commercial or financial relationships that could be construed as a potential conflict of interest.
